# Mediating role of wellbeing among organizational virtuousness, emotional intelligence and job performance in post-pandemic COVID-19

**DOI:** 10.3389/fpsyg.2023.1105895

**Published:** 2023-01-27

**Authors:** Muhammad Ali Arshad, Darwina Arshad, Nazlina Zakaria

**Affiliations:** School of Business Management, College of Business, Universiti Utara Malaysia, Sintok, Kedah, Malaysia

**Keywords:** wellbeing, organizational virtuousness, emotional intelligence, employee performance, Smart-PLS, COVID-19

## Abstract

It is devastating to people’s mental and emotional health to be exposed to the COVID-19 pandemic and the multifaceted response strategies are required to curb it. As a result of social distancing and self-isolation, people have faced many challenges in their lives. The suffering is even greater at the workplace where the employees are working with the fear of getting exposed to the virus and its new variants which is adversely affecting their wellbeing. This study explores and tests a model that extends the wellbeing research across organizational settings and targets the crucial factors that lead to job performance improvement even in the post pandemic COVID-19 situation. To improve both in-role performance and extra-role performance behaviors in the Pakistan banking sector, organizational virtue (also known as organizational virtuousness) and internal virtue (also known as emotional intelligence) are examined. Data were collected from the 416 bank employees using disproportionate stratified sampling technique. In the bank sector of Pakistan, wellbeing was identified as the key psychological factor that relates the in-role performance and extra-role performance to internal and organizational factors. Research findings also determined that conceptualizing subjective wellbeing in the context of work is more meaningful in understanding its relationship with the workplace variables than the general or global subjective wellbeing.

## Introduction

When people are exposed to the COVID-19 pandemic and the many different reaction tactics implemented to stop it, it can have a catastrophic effect on their mental and emotional health. Employees have said that they have struggled with various issues throughout their lives due to social isolation and self-isolation. It is necessary to recognize, investigate, and address these consequences in order to deal with them in order to minimize detrimental effects in order to ensure the wellbeing of persons, particularly those who are in the job. Notably, the emergence and spread of the COVID-19 virus negatively affected wellbeing. Similarly, the consequences of the COVID-19 pandemic on work place wellbeing and job performance are even greater. The pandemic generated a wide range of emotions, thoughts, and reactions worldwide (Mahmood et al., 2022). According to the global happiness report 2021, “The workplace happiness has declined. Happiness and life satisfaction saw one of the largest declines during the pandemic, along with mental and physical health, together with more modest declines in meaning in life and overall flourishing. The frequency of positive emotions has fallen, and of negative emotions has risen, with the increase in negative emotions much higher than the reduction in positive emotions” (Helliwell et al., 2021, p. 34). It is important to keep in mind that Pakistan, as a developing country, is vulnerable to COVID-19 (Atif and Malik, 2020). The risk of a poor health system makes the residents apprehensive. Through risk communication and engagement, the government is strategically aligning the expanded scope of community ownership and understanding in the population; however, despite the efforts of the government, the situation still appears to be very austere, and it has even affected the psychological health of employees and employers.

In the country’s economic development, the banking sector plays a significant and valuable role in supporting economic growth. The banking sector has gained more significance in developing countries like Pakistan due to less developed money and capital markets and an uncertain economic environment (Karim et al., 2022). To accumulate capital for various sectors of the economy needs, the government of these countries relies on the banks as a significant source of funds because the inflow of funds in the form of foreign capital has decreased due to several factors such as terrorism and mistrust between foreign donor and government (Singh and Kapuria, 2021). More recently, the outbreak of the COVID-19 pandemic has led to unanticipated changes in the outlook of the economic system around the world. The uncertainty caused by COVID-19, particularly in developing countries, has caused lower economic growth and affected the banking sector by reducing foreign investments and credit growth (Barua and Barua, 2021). The prevailing condition has thus provided challenges for the management of the banks in finding ways of improving growth and profitability under this uncontrollable external environment.

One of the vital internal factor contributing to banking organizations’ financial performance is employees’ job performance. Every organization relies considerably on individual employee performance to gain high organizational performance (Gazi et al., 2022). According to O’Connell (2022), the profitability of banks can be increased through the high performance of employees, which will be beneficial in the stiff competition of the banking industry. However, the banking sector is facing the problem of decreasing employees’ job performance (Inayat and Jahanzeb Khan, 2021). It is crucial for the survival and sustainability of a bank that its employees are high-performing individuals who can meet the expectations of their management and customers. The aggressive nature of the competition and the globalization of the markets, have compelled the banking institutions to implement unique strategies for enhancing their own internal performance in order to be able to effectively compete (Moghadam and Salamzadeh, 2018). Due to these factors to keep up with the competition and maintain their market share, the banking institutions must be aware of the challenges faced by their workforce and actively develop strategies that will enable their staff to function at the highest possible level despite the challenging conditions.

Wellbeing in terms of “Happiness,” as an academic concept, has sparked formidable interest for the researchers from varying fields as organizational behavior, sociology, psychology, and economics in recent years. Of note, plethora of research has been done on this concept in the diversified academic fields with focus on studying its relationship with performance in workplace setting. With growing focus on positive psychology (Seligman, 2002), it has been labeled “happy-productive worker thesis,” i.e., individuals and groups with feelings of higher levels of wellbeing perform better in their jobs than do those with lower wellbeing. Support for this “commonsense theory” can be traced to the seminal Hawthorne studies (Roethlisberger and Dickson, 2003), which concluded that higher levels of job-related performance were attributed to happy workers, compared to their unhappy counterparts (Wright and Cropanzano, 1997; Wright et al., 2002; Peiró et al., 2019).

Notwithstanding the general support for the presumed “happy-productive worker” link, there still remains lack of consensus on the conceptualization. Empirical researchers are hard-pressed to establish a close link between employees’ happiness and their performance (Hosie and Sevastos, 2009; Taris and Schreurs, 2009; García-Buades et al., 2020). Infact, in two studies of the relationship among individual-level job satisfaction (tapping the affective dimension of wellbeing) and “performance,” their relationship was determined at 0.14 (Vroom, 1964), 0.17 (Iaffaldano and Muchinsky, 1985). While, on other hand, a prominent meta-analysis revealed that when several aspects of job satisfaction (affective and cognitive) are formed into a composite construct of happiness, there was a prominent revised relationship of r 0.30 among happiness and job performance (Judge et al., 2001). In similar work, Warr and Nielsen (2018), in their meta-analysis, study various kinds of job-related and context-free wellbeing with work performance in form of extra-role and in-role behavior. They compiled the outcomes of analysis as positive but low. In sum, it can be inferred that decades of work still unfruitful in providing strong theoretical and empirical evidences for understanding the happiness and performance link at the workplace, that is also considered as the “Holy Grail” of industrial and organizational psychology (Wright and Cropanzano, 2004).

It is clear that employee wellbeing has a crucial role to play in improving performance behaviors because it is well-known that “happy workers are also productive workers.” Employee wellbeing is considered crucial not only for employee performance but also for the profitability of the bank as a whole. Organizations in Pakistan, especially the banking sector, have largely overlooked employee wellbeing as one of their key challenges (Gulzar et al., 2020; Bhardwaj et al., 2021; Tajpour et al., 2021). As the banking sector in Pakistan is facing an extremely competitive environment today, it is imperative that employers take good care of their employees in order to stay competitive. To increase the productivity of the employees, banks must identify all the factors that can contribute to the wellbeing of the staff at work in order to increase the level of productivity at the workplace the normal operations period but also in the post pandemic era.

This study explores the relationship between OV and EI and EMP at five significant Pakistani banks, with a focus on the potential mediating function of work-related SWB. Based on the results of this study, the theoretical contribution of the study could be applied to the field of human resource management, in the area of positive organizational psychology and to continue to progress the study on employees’ subjective wellbeing as it relates to employee performance at work as a way to enhance the understanding of organizational behavior. This study provides insights that can assist management in the Pakistani banking sector in designing their organizational policies and practices to create an environment conducive to make employee feel happy to perform well.

From the critical literature review on wellbeing, happiness and job satisfaction in relation to job performance from various fields following gaps are identified that warrant urgent attention to solve this so-called “holy grail of the organizational psychology” in business management. Firstly, though, previous works found modest assistance for Happy-worker productive worker thesis (Judge et al., 2001; Hosie et al., 2012; Kabene and Baadel, 2020). However, one of the discrepancies of the thesis is that it does not highlight measures to enhance employee’s wellbeing to increase their performance behaviors (Kaur et al., 2020), as employees cannot be happy by themselves. Thus it does not highlights the key predictor of employee wellbeing in boosting their job performance. In addition, earlier research has suggested that both the external and internal factors should be included to gain a deeper understanding of the mechanisms that connect these variables with the performance, previously ignored to gain a deeper understanding of their relationships.

Secondly, researchers have given largely insufficient attention to distinct dimensions of job performance in studying the relationships between wellbeing and job performance (Hosie and ElRakhawy, 2014). Thirdly, there is scarcity of empirical work on wellbeing in organization settings (Merdeka et al., 2020), especially in Asian context (Sender et al., 2020). Therefore, expanding the work into other sectors of the underdeveloped economy that place great importance on employee wellbeing, such as the banking industry, is imperative.

Lastly, and most importantly, terms such as “wellbeing,” “Happiness,” and “Job satisfaction” have been synonymously and interchangeably used in the literature creating confusion regarding their differentiation. Moreover, to date, the majority of the scholars from various fields failed to provide a universal single definition and operationalization of these related terms (Wright, 2014; Al Suwaidi, 2019; Mohammed and Abdul, 2019; Joanna and Jerzy, 2020; Tuzovic and Kabadayi, 2020). Similarly, there is no single acceptable measurement exist to measure the complete aspect of wellbeing, happiness or job satisfaction. Although various instruments are available but they measure either one of the facets not the complex multidimensional aspect of these construct. Saks and Gruman (2014) mention certain issues. The first disquiets the lack of agreement on its definition and the other is concerned with its measurement. It is argued that most of the problems/issues are embedded in the definition and operationalization of the construct of happiness and performance. So far, very little work has been done on defining a single concept of wellbeing in terms of workplace that can be universally agreed upon. Therefore, it is extremely important to extend the research in this field.

The purpose of this study is to identify and address the gaps in the previous literature and to find a way forward for the direction of the research. In order for the researcher to accomplish this, it conceived up and validated a research model that is founded on both theoretical and empirical evidences.

## Literature review

### Subjective wellbeing (work-related model)

The literature review as already discussed in the previous section revealed that, terms such as “subjective wellbeing,” “wellbeing,” “job satisfaction” and “Happiness” have been synonymously used. Wellbeing has been operationalized by a variety of complex constructs touching one of the element of happiness, for example, affect, satisfaction, work engagement, quality of working life (Cropanzano and Wright, 2001; Hosie et al., 2007; Sender et al., 2020) and more commonly as job satisfaction in the work performance link (Wright, 2005). Even though, when various investigations utilize the same construct, the measurement scale was different. Diener (1984) took a more rational approach to the problem. He proved that virtually all scientific explanations of happiness could be boiled down to three key points that set them apart. According to Diener (1994), happiness is, first and foremost, a uniquely individual experience contingent on each individual’s point of view. In addition to both affect (positive and negative), a greater number of positive emotions in combination with a relatively lower number of negative emotions is a significant component in determining levels of happiness (Argyle, 1987). As a final point, happiness is a universal evaluation. As a result, a person’s entire life is taken into account when judging them. He added that happiness exhibits consistency over time (Myers, 1993; Myers and Diener, 1995). Overall, Diener classified subjective wellbeing (SWB) as the term for happiness. He explained that the evaluation of a person’s life on a cognitive and emotional level is subjective wellbeing. Most academician recognized this happiness paradigm as the hedonic wellbeing model, or the hedonic wellbeing theory. As a matter of fact, happiness is defined as the wellbeing that can be experienced by an individual (Ryan and Deci, 2001). Diener et al. (1998) found that the absence of bad moods, the existence of good moods, and the satisfaction with life can significantly impact happiness levels.

The other prospect given into contrary to hedonic is of eudemonia wellbeing/happiness. The eudemonic perspective shows engagement in activities that foster human growth, such as autonomy, personal growth, self-acceptance, life purpose, mastery, and positive relatedness, as essential to wellbeing (Ryff and Keyes, 1995; Ryff, 2013). While Ryff’s (2013) model centers on eudemonic elements, researchers stated that hedonic presents an important part in wellbeing. On the hedonic SWB conception and its measurement scale for assessing an individual’s level of happiness, the vast majority of academics have reached a consensus (Lent and Brown, 2008). In the same way, some studies on wellbeing in term of happiness had been conducted from a hedonistic point of view (Lorente et al., 2019). Although the subjective wellbeing (SWB) framework is generally thought of as context-free, In the workplace wellbeing paradigm, there has only been a relatively limited amount of research undertaken (Houge Mackenzie and Hodge, 2020).

**FIGURE 1:**
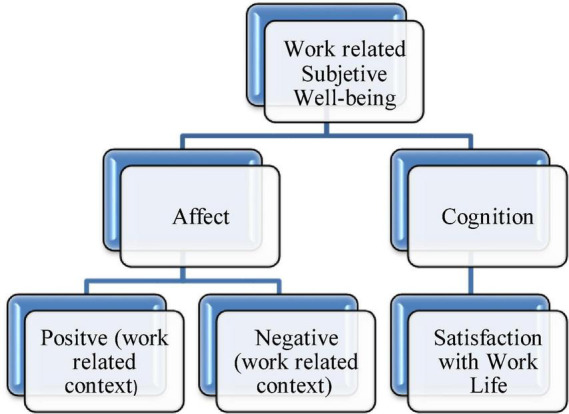
Wellbeing conceptualization.

By employing the hedonic paradigm in the context of the workplace, the study presented employee wellbeing model referred as “work-related subjective wellbeing” or “employee subjective wellbeing,” respectively (refer to Figure 1). It is defined as “employee affective and cognitive appraisal of work-life,” The definition is line with the suggestions from the previous scholars researchers (such as Bakker and Oerlemans, 2011). An employee’s emotional response and feelings at work, are considered as the affective evaluation, whereas, the cognitive evaluation and judgment refers to what employees think about their work or employment.

The justification of proposing the new definition lies in the fact that this definition considers both the cognitive and affective component in forming the composite employee wellbeing construct that has been identified in the previous literature as one of the reasons for its misinterpretation by tapping only one of the components/facet, i.e., either affective or cognitive. Another plausible justification for proposing this definition is that the operationalization of the complex wellbeing construct has now become simpler and easily understandable among the scholar of same interest from various field. It proposes that wellbeing as a multidimensional construct, will be measured as a high order composite wellbeing, with an affective and cognitive component on a single order. These arguments are based on the recommendations and empirical evidences from the previous scholarly works. For example, various attitude scholars suggested that to achieve construct (wellbeing) correspondence with job performance, considering both component (affective and cognitive) as a whole is the most appropriate approach (e.g., Norman, 1975, Fazio and Zanna, 1978; Bagozzi and Burnkrant, 1979; Schleicher et al., 2004). Similarly, Judge et al. (2001), concluded that when different facets of job satisfaction (affective and cognitive) are formed into the composite measure, there is a notable corrected correlation of r 0.30 among satisfaction and performance. Moreover studies on human brain also suggested that emotional and cognitive regions influence one another *via* a complex web of connections in ways that jointly contribute to behavior (e.g., Okon-Singer et al., 2015). Similarly some scholars have also demonstrated the neural integration of emotion and cognition in the brain *via* imaging (Shackman et al., 2011; Raz et al., 2012). As a result, the proposed definition is consistent, not only conceptually and intellectually, but also experimentally, with the earlier thoughts and research.

The term “wellbeing” refers to a state of mental and physical fitness that can be affected by a variety of factors, depending on the setting. Further, studies have also conducted on the outcomes of wellbeing. As discussed earlier, limited studies have shown that there is a positive correlation between an employee’s level of happiness and their level of performance on the job (e.g., Zheng et al., 2015; Krekel et al., 2019; Mardanov, 2020, 2021). Several different antecedents of happiness have been uncovered by other researchers. These antecedents include emotional intelligence (Carmeli, 2003), organizational rewards (Nthebe et al., 2016), perceived organizational support (Ahmed and Nawaz, 2015), organizational virtuousness (Asad et al., 2017), and personality (Higgs and Dulewicz, 2014). It has been stated by Nanda and Randhawa (2019) that, in order for organizations to manage their employees’ emotional states at work, they must create an environment in which workers can feel happy while at work and thus be able to manage their emotional states effectively. Even though wellbeing is a highly relevant variable, it has, for the most part, been ignored in work place context. Furthermore, very few studies have investigated the question of whether or not wellbeing mediates the relationship between the individual difference in both IRP and ERP by identifying several factors in the workplace that triggers happiness (Haryono et al., 2019).

### Job performance

When it comes to reaching different results and accomplishments, organizations place a strong emphasis on their employees’ job performance. It was Campbell (1990) who first formulated the concept of job performance and describe job performance as “synonymous with behavior” (pg. 40). This is something that people really do and can be observed. By definition, it includes behaviors or actions that are related to the organization’s goals. Work that an organization hires people to do and expects them to accomplish successfully is referred to as performance. Campbell et al. (1993) further stated that the only action that can be restrained could be considered as performance. Besides, this behavior was pointed out as manageable and will be drawn toward the organization’s objectives (Campbell et al., 1993). It is a widely held belief that employee performance on the job cannot be reduced to a single dimension, even though different academician has come up with varied definitions of what constitutes good job performance. Therefore, it ought to be understood and conceived of as a variable comprising various behaviors in their many manifestations.

Although many attempts have introduced different performance frameworks, Campbell’s definition of performance has been widely accepted as the basic definition of job performance Scholars have put forward different dimensions of job performance based on the multi-dimensional nature of human behavior, however, A closer inspection reveals that they only focus on two or three unique dimensions, namely In-role performance (IRP) or task and Extra-role performance (ERP) or contextual performance.

The notion of task performance was first introduced by Williams and Anderson (1991) and then Borman and Motowidlo (1997). Based on the behaviors they observed, they classified work performance into two categories: task performance and contextual performance. Task performance, on the other hand, is defined as behaviors specific to a job, which are formal and fundamental to the job as a whole. While, employee behaviors are normally categorized as contextual performance when they have the potential to impact the whole context in which they performs work as well as the overall psychological and organizational context in which it exists.

In addition, a review of the literature in the field confirmed that some other labels for in-role performance had also been used previously. These include task behavior (Murphy, 1989); job-specific task proficiency (Campbell et al., 1990; Griffin et al., 2007; Wisecarver et al., 2007); technical proficiency (Lance et al., 1992; Campbell et al., 2001); task performance (Borman and Motowidlo, 1993) in-role performance (Bakker et al., 2004; Maxham et al., 2008) and more recently individual work performance (Koopmans et al., 2011). The ERP dimension had also been interchangeable with several other labels in the literature. These include: contextual performance (Borman and Motowidlo, 1993);extra-role performance (Maxham et al., 2008); citizenship Performance (Organ, 1997; Organ and Paine, 1999; Coleman and Borman, 2000); non–job-specific task proficiency (Campbell, 1990); organizational citizenship behavior (Smith et al., 1983; Williams and Anderson, 1991); non-prescribe behavior (Orr et al., 1989); interpersonal relations (Murphy, 1989); organizational spontaneity (George and Brief, 1992) among others.

Concluding, all of these terms overlaps each other conceptualization and are mostly used interchangeably through the performance literature. The fact that managers or employers use ERP in addition to IRP when evaluating an employee’s performance is one of the main justifications for distinguishing the two types of job performance. Further, because both of these factors contribute to the success of the companies in their own different ways, it is essential to investigate factors affecting both the performance, even though previous researchers have largely neglected this aspect. By focusing on the factors that affect employee wellbeing, this study hopes to close this gap and improve the performance of the organization as a whole.

### Organizational virtuousness

In organizational literature, the concept remained neglected by scholars and did not draw the practitioner’s attention. Nevertheless, due to emerging financial and moral crises around the globe, prevalent business press and business community tend to believe that nurturing virtuousness both at the organizational and individual level can enhance efficiency and performance (Cameron, 2010; Rego et al., 2011; Magnier-Watanabe et al., 2020) therefore, virtues should be considered in the agenda of business and management researches. Positive organizational scholarship is where the concept of organizational virtuousness (henceforth abbreviated as OV) first emerged. OV has been formally defined as “individuals’ actions, collective activities, cultural attributes, or processes that enable dissemination and perpetuation of virtuousness in an organization” (Cameron et al., 2004, p. 768). It is characterized as the presence of an atmosphere in which trust, humanism, forgiveness, optimism, and integrity flourished, maintained, and spread across the members of the organization (Cameron et al., 2004). Employees may develop a more optimistic outlook on their jobs due to their exposure to the organization’s core values, which, in turn, will likely affect their attitudes and behaviors (Rego et al., 2010).

As a concept, OV has received limited attention since its origin (Cameron et al., 2004), and only a small number of empirical studies have examined its consequences and determinants. Although research on this topic is sparse, some researchers have discovered that organizational and individual-level effects can be brought about through the perception of OV (for example, Bright et al., 2006; Cameron et al., 2004; Rego et al., 2015; Ahmed et al., 2018). It is argued that employees are likely to represent great performance in their job and put most of their efforts for the organization and their work when they experience their organization to be good and virtuous (Magnier-Watanabe et al., 2017). Moreover, when employee perceive organizational support (in the form of virtuous environment) they will be motivated to be grateful to their employers by engaging in ERP and IRP (Ahmed et al., 2018). Similarly, when employees perceive OV, they form emotions, organizational image, and self-construal, making their relationship stronger with their employer (Rhee et al., 2006).

Though some scholars have provided empirical evidences of OV significant direct relationship with the job performance (Ahmed et al., 2018; Pires and Nunes, 2018; Sun and Yoon, 2022) and also with the wellbeing (Rego et al., 2011; Nikandrou and Tsachouridi, 2015; Asad et al., 2017; Singh et al., 2018; Salas-Vallina, 2020), however, only a handful number of studies have focused on the psychological mechanism through which OV influences workplace outcomes and warrants further investigations (Ahmed et al., 2018; Dubey et al., 2020). Therefore, this study has considered the wellbeing as mediating mechanism for linking OV and job performance in the workplace to bridge this gap.

### Emotional intelligence

The basic idea of Emotional Intelligence (henceforth abbreviated as EI) can be found in the noticeable study by Thorndike (1920). According to Thorndike (1920), intelligence is basically the junction of three aspects, which are abstract intelligence (capability to manage and understand ideas), mechanical intelligence (associated with real objects) and social intelligence (related to people). He further defined social intelligence as “the ability to understand and manage men and women, boys and girls—to act wisely in human relations.” (p. 228). Later, Salovey and Mayer (1990) first defined EI and gave the true meaning of EI in their developed theory which is also called the ability model. Emotional intelligence is comprised of four different competencies that are all intertwined with one another. These competencies are: recognizing one’s own feelings as well as the feelings of others; using emotion to facilitate thought; recognizing emotional information, and controlling one’s own feelings and those of others (Mayer et al., 1990). According to the notion of EI, workers who have a high level of EI will have better success in their personal and professional lives (Carmeli, 2003).

There is considerable evidence in favor of EI, showing that EI is more critical than job-specific (technical) skills and knowledge or IQ. Brotheridge and Grandey (2002) regarded EI as twice as important as IQ and technical expertise and four times as important in overall success. Sternberg (1996), in another analysis, stated that IQ only explains 4 to 10 percent of success at work. Further, EI is double as important as intellectual intelligence, and IQ only explains twenty percent of the factors that cause life success (Goleman, 1996), whereas emotional intelligence accounted for 80% of the determinants of individual success (Martinez-Pons, 1997; Boyatzis et al., 2000). Moreover, Dulewicz and Higgs (2000) found EI as a vital success construct that directly contributed more than managerial intelligence and intelligence quotient (IQ) in job performance. Therefore, EI has become the focus of the interest of several researchers in examining the EI and job performance relationship.

In a famous study conducted by Wong and Law (2002), who used empirical research as a basis for the study on emotional intelligence, discovered a significant correlation between EI and outcomes related to the workplace. In addition, Gong et al. (2019) and Prentice (2019) have demonstrated that EI influence a number of attitudes and positive behaviors related to the job, including job satisfaction and job performance. It is argued that in contrast to someone with a lower EI score, an individual with a high EI score would be expected to be better capable of comprehending, perceiving, and managing their emotions in a way that might affect their success at work. This is why Mayer et al. (2000) suggest that EI may be an important variable in determining job performance. This is because employee with high EI are better able to understand, perceive, and have the ability to regulate their emotions in a way that contributes to their success in their careers.

More importantly, large number of studies have empirically determined EI as an important construct for increasing job performance (i.e., Sendaro and Baharun, 2020; Alheet and Hamdan, 2021; Furnham and Treglown, 2021; Miao et al., 2021) and also the wellbeing of the employees (i.e., Akhtar et al., 2017; Fida et al., 2019; Xu et al., 2021; Peláez-Fernández et al., 2022; Sha et al., 2022). However, limited studies have explored the mechanism by which EI leads to better IRP and ERP. Thus, this study aims to address this gap by considering wellbeing as a key intervening variable that is proposed to cause EI enhancing the job performance.

### Theoretical underpinning

The Affective Events Theory (AET) theory gives us a good theoretical reason to look into the mediating processes *via* which background information and personality traits influence behavior in the workplace. AET provides an extremely useful theoretical framework for the investigation of these mechanisms. The purpose of this is to understand how people behave in various situations in order to better predict their behavior. From a theoretical point of view, there is a possibility that, within the context of AET, a work-related SWB may play an important role in mediating between dispositional factors (such as personality traits or emotional intelligence) and external factors (Organizational Virtuousness) in the relationship between performance behaviors and performance factors. A work-related SWB is one that is related to the workplace (i.e., the use of IRP or ERP) and therefore has a greater impact. Behavioral, affective, and emotional aspects may be crucial intervening explanations for how people behave at work and how these elements influence their behavior (Devonish, 2016). An affective state is proximal to affective events, which are important to wellbeing on the basis of AET. The impact of the effect on work behavior is determined by the impact of the effect on job attitude (Weiss and Cropanzano, 1996). As the holistic concept of wellbeing ties into cognitive and affective constructs (Greenidge et al., 2014; Devonish, 2016), this study examines how an external factor (organizational virtuousness) may contribute to IRP and ERP since virtuousness is seen as a cognitive and affective construct.

In this study, we demonstrate that according to the assumptions of AET, work-related SWB may enhance or hinder job performance (i.e., IRP and ERP depending on both cognitive and affective dimensions of the employee), considering observed levels and quality of employees (EI). This is true regardless of whether or not the employee is engaged in their work (Devonish, 2016). As a result, the study’s hypothesis claimed that work-related subjective wellbeing would mediate between individual factors (EI) and job performance. In addition to this, Organizational support theory (OST) is another theory that supports the relationship between the study’s variables. In accordance with the tenets of the OST, an employee is expected to demonstrate positive behavior to demonstrate gratitude for their benefits from their employer. This is because the employee feels obligated to show gratitude (Eisenberger et al., 1986). According to this presumption, the assistance provided by employees comes from working in an environment that exemplifies virtue. When people have the impression that they are being supported, they experience increased levels of happiness and satisfaction, which contributes to employees’ strong sense of wellbeing. Because of this wellbeing, the employee will have the feeling that they are compelled to provide favorable treatment to the organization in return for the favorable treatment they have got, and they will participate in IRP and ERP in order to help the business realize its goals.

### Research framework

The following conceptual model is presented in light of the research conducted for this study, which combines theoretical underpinnings, empirical findings, analytical support, and an examination of relevant literature. All of these elements help form the model’s foundation.

### Research hypothesis

**FIGURE 2:**
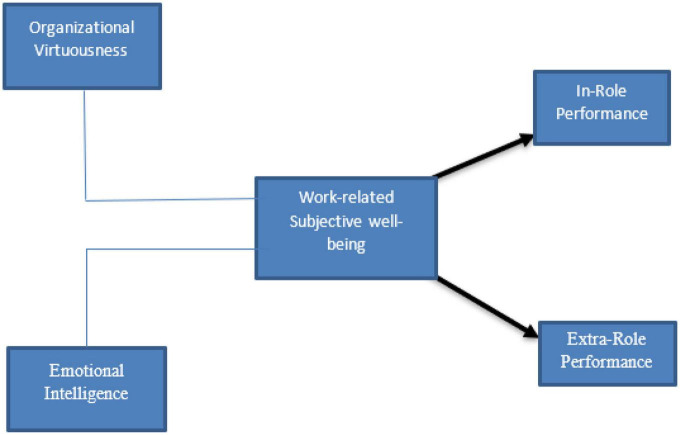
Research framework.

On the basis of the literature review and theoretical underpinning discussed in the previous sections, the following hypotheses are formulated as shown in Figure 2:

H1: There is a mediation effect between OV and IRP in relation to work-related SWB.

H2: There is a mediation effect between OV and ERP in relation to work-related SWB.

H3: There is a mediation effect between EI and IRP in relation to work-related SWB.

H4: There is a mediation effect between EI and IRP in relation to work-related SWB.

## Methodology

The findings of this study are derived from survey responses from 416 bankers who were chosen using disproportionate stratified sampling. Data was gather from the employees working in management positions (such as branch manager and branch operations manager) at the branches of Pakistan’s five largest banks namely, Habib Bank Limited (HBL); MCB Bank Limited (MCB); National Bank Limited (NBP); United Bank Limited (UBL); and Allied Bank Limited (ABL), which were 7,282 (State Bank of Pakistan, 2018). The table developed by Krejcie and Morgan (1970) was utilized in the research to estimate the suitable size of the sample from the population of 7,282 (State Bank of Pakistan, 2018). However, at the time that the data was collected, the population was down to 6,514 as a result of instructions from the State Bank of Pakistan (SBP) to banks to operate with a smaller number of branches and the closure of branches in regions with a high incidence of COVID-19 virus. In addition, the researcher handed out 550 questionnaires to make up for the lower response rate, which was 40 percent (according to Umrani and Mahmood, 2015) and 51.5 percent (according to Ahmed, 2017) in earlier research carried out on the banking industry of Pakistan. Demographic information about the respondents and their respective organizations, including the respondent’s bank, gender, age group, the highest level of education, years of experience, Official Cadre, and functional title/designation is given in Table 1.

**TABLE 1:** Descriptive analysis of demographic data.

Demographic variables	Category	Frequency	Percentage (%)	Cumulative percentage (%)
Bank	HBL	92	22.1	22.1
	MCB	88	21.2	43.3
	UBL	104	25	68.3
	ABL	76	18.3	86.5
	NBP	56	13.5	100.0
Gender	Male	342	82.2	82.2
	Female	74	17.8	100.0
Age	21–30 Years	110	26.4	26.4
	31–40 Years	168	40.4	66.8
	41–50 Years	122	29.3	96.2
	51–60 Years	16	3.8	100
Education	Ph.D.	0	0	0
	Masters	236	56.7	56.7
	Bachelors	164	39.4	96.2
	Diploma/Associate degree	16	3.8	100
Experience	1–5 Years	92	22.1	22.1
	6–10 Years	174	41.8	63.9
	11–15 Years	120	28.8	92.8
	16 Years and above	30	7.2	100.0
Cadre/Grade	OG–1	38	9.1	9.1
	OG–2	128	30.8	39.9
	OG–3	100	24	63.9
	OG–4	142	34.1	98.1
	OG–5 and above	8	1.9	100.0
Functional title	Branch manager	266	63.9	63.9
	Branch operation manager	150	36.1	100

### Measurement scales

A five-point Likert scale was used to acquire the respondents responses and validated and well-established. In order to measure the variables following well established and validated scales are used. The 15-item measure that Cameron et al. (2004) established for OV’s evaluation was used. As, there is not yet a single scale capable of measuring both the emotional (including both positive and negative sensations) and the cognitive aspects of wellbeing, subjective wellbeing was examined using two distinct scales (satisfaction with life). In addition, in order to get a sense of the respondents’ levels of SWB in relation to their work, we asked them to frame their comments in terms of their work. This allowed us to get a better sense of the respondents’ SWB. The 12 item Scale of Positive and Negative Experience (SPANE) that was developed by Diener et al. (2010) was used in this concern to measure the affective wellbeing, and the 5-item SWLS (Satisfaction with Life scale) that was developed by Diener et al. (1985) was adapted to measure the cognitive component of wellbeing. Both of these scales were developed by Diener et al. (2010). Diener et al. (2010) are responsible for the development of both of these scales. In addition, the Wong Law EI Scale (WLEIS) (Wong and Law, 2002) was applied to individuals to ascertain the amount of EI they possessed. In addition, the IRP was evaluated utilizing a scale that consisted of 7 items and was established by Williams and Anderson (1991). In contrast, the ERP was evaluated utilizing a scale that consisted of 8 items and was developed by Koopmans et al. (2013). In this investigation, trustworthy and valid measurements were used to establish the variables that were to be introduced, and the measures themselves were all regarded as acceptable and useful based on the findings of the study.

## Analysis and results

The study used PLS-SEM technique for measuring path model, which included both the structural and the measurement models, to test the hypothesis. The reason of using PLS-SEM is because the study model consisted both reflective and formative construct, therefore, on the basis of recommendation of prominent scholar, (Sarstedt et al., 2021), the data was analyzed with Smart-PLS 3.2.8. In addition, in order to evaluate both reflective and formative measurement models, the authors of this study utilized a two-stage methodology that included the first-order reflective measurement model and the second-order reflective-formative hierarchical model. This methodology was suggested by the authors of the previous study of Becker et al. (2012).

### Assessment of measurement model

It is important to stress that as this study was conceived as a construct of both reflective and formative components, it is important to focus on each separately. We chose to use PLS-SEM, as recommended by prominent scholars in the field of measurement (Sarstedt et al., 2017a,b). This section examines the second-order formative measurement which combines first-order reflections along with second-order (higher-order) formative measures.

### Assessment of reflective measurement model (first order)

**FIGURE 3:**
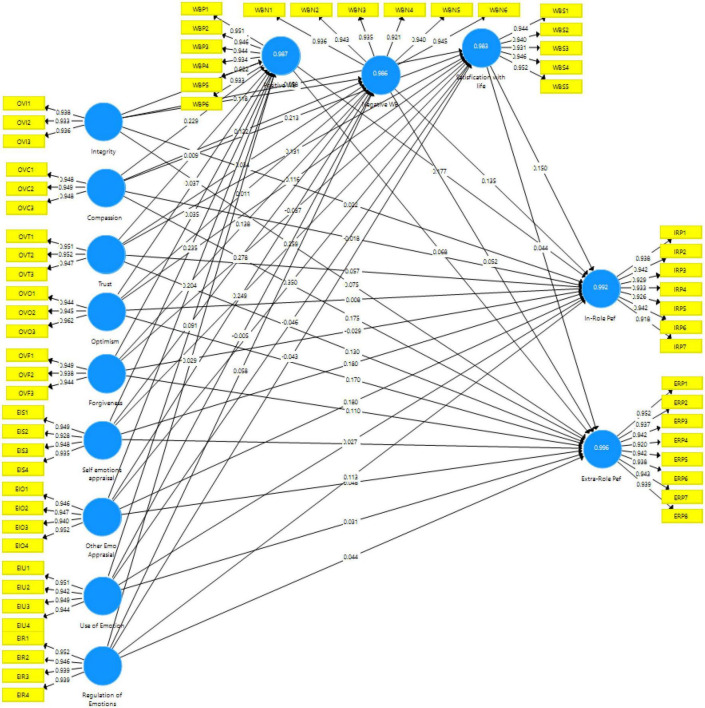
First order measurement model.

Validity and reliability were determined using the first-order reflective measurement model. In order to accomplish this goal, the research investigated the reliability of individual items, as well as the consistency of the dependability within itself, convergent validity, and discriminant validity. In Figure 3, you can see an image depicting the PLS algorithm diagram, which was used in order to assess the measurement model. Additionally, the results of their evaluation can be found in Table 1 as well as a report on their evaluation process. Each construct’s outer loading was examined as part of the work undertaken to assess its dependence on individual items. This was done to determine whether those outer loads corroborated with the dependability of individual items (Hair et al., 2014). Hair et al. (2014) conducted a study detailing that a maximum value of outer loading was acceptable as determined by the maximum value of 0.700, which was determined by the research. As a direct consequence of this, the item loadings in the current study ranged anywhere from 0.822 to 0.962. Furthermore, we investigated the composite reliability (CR) coefficient and Cronbach’s alpha (CA) coefficient to determine the degree of internal consistency dependability as proposed by Peterson and Kim (2013). This CA result exceeded the threshold value of 0.70, which was 0.872–0.912. Additionally, the CR values were 0.876–0.916, which are higher than the cutoff value of 0.7 (Hair et al., 2010). This resulted in significant internal consistency reliability for every construct in the study. Thirdly, CV was analyzed using the values obtained from the Average Variance Extracted (AVE) statistic. According to the recommendations made by Chin (1998), an AVE value that is larger than 0.5 should be regarded as satisfactory. According to the suggestions made by Chin (1998), the findings showed that the convergent validity was good because the AVE was greater than 0.50 on their important constructs. In conclusion, the current research utilized the Fornell-Larcker Criterion in order to evaluate the discriminant validity of the results (Hair et al., 2010). Fornell and Larcker (1981. p. 11) recommend that, for all reflective constructs, “the square root of the AVE (diagonal) needs to be higher than the correlations (off-diagonal) in order to achieve sufficient discriminant validity.” In Table 2, it appears that the study met the Fornell-Larcker Criterion as well.

**TABLE 2:** Reliability and validity.

Construct	Loadings	CA	CR	AVE
Integrity	0.878–0.916	0.911	0.916	0.84
Compassion	0.833–0.938	0.9	0.907	0.799
Trust	0.847–0.951	0.899	0.902	0.703
Optimism	0.844–0.962	0.875	0.879	0.687
Satisfaction with life	0.840–0.931	0.88	0.885	0.679
Forgiveness	0.844–0.938	0.872	0.876	0.674
Self-emotions appraisal	0.897–0.945	0.91	0.915	0.838
Extra-role performance	0.838–0.938	0.902	0.907	0.832
Use of emotion	0.842–0.944	0.898	0.903	0.825
Regulation of emotion	0.846–0.939	0.901	0.906	0.83
Positive wellbeing	0.822–0.944	0.903	0.91	0.836
Negative wellbeing	0.836–0.945	0.899	0.905	0.829
Other emotion appraisal	0.847–0.940	0.912	0.916	0.84
In-role performance	0.825–0.0933	0.879	0.883	0.74
Trust	0.847–0.951	0.899	0.902	0.703

### Assessment of formative-measurement model (second order)

**FIGURE 4:**
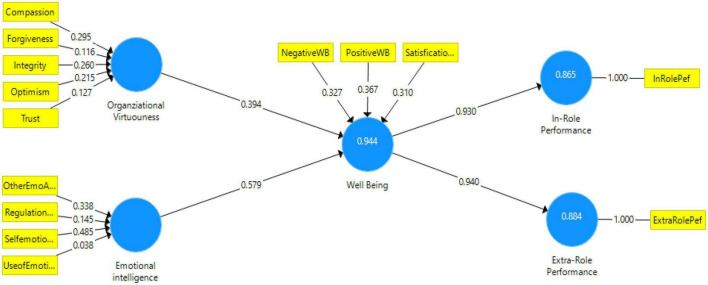
Second-order structural model.

Measuring the formative construct in conjunction with the measurement model, Hair et al. (2014) developed a set of criteria related to the overlap and significance of outer weights as well as the collinearity issue. Figure 4 depicts the level of the partial least squares diagram that corresponds to the second-order formative measurement model. Additionally, the results of the assessment of the formative measurement model are presented in Table 3, which shows that for the constructs within the model, the outer weights have been found to be significant, along with the *t*-values associated with the formative constructs. Despite the fact that the emotional intelligence indicator known as “use of emotion” was not relevant (*t*-value = 0.338) to the formative construct of emotional intelligence. Despite this, we will continue to use this signal because the outer loading it contributes is significantly more significant than 0.50. In addition, the VIF numbers were used to investigate the collinearity issues. This was done to determine whether or not the formative indicators have a strong correlation with one another. A VIF value of 5 or higher indicates that there may be a problem with collinearity (Hair et al., 2014). As illustrated in Table 3, the results of this study indicate that for each formative indicator, the VIF values fell below the threshold value of 5 in light of the findings. Consequently, all the VIFs for the constructions did not mention any issues with multicollinearity appearing in the VIFs of the constructions.

**TABLE 3:** Fornell-Larcker Criterion.

	1	2	3	4	5	6	7	8	9	10	11	12	13	14
Compassion	0.978													
Extra-role pef	0.835	0.939												
Forgiveness	0.867	0.883	0.944											
In-role pef	0.888	0.890	0.875	0.932										
Integrity	0.916	0.885	0.773	0.881	0.936									
Negative WB	0.778	0.791	0.679	0.790	0.780	0.784								
Optimism	0.872	0.785	0.760	0.877	0.672	−0.770	0.950							
Other emo appraisal	0.775	0.888	0.872	0.789	0.675	−0.786	0.912	0.946						
Positive WB	0.882	0.791	0.775	0.791	0.678	−0.792	0.777	0.665	0.938					
Regulation of emotions	0.775	0.872	0.874	0.788	0.723	−0.785	0.721	0.644	0.684	0.786				
Satisfaction with life	0.879	0.789	0.771	0.690	0.676	−0.792	0.717	0.636	0.629	0.685	0.943			
Self emotions appraisal	0.875	0.889	0.874	0.790	0.811	−0.794	0.656	0.641	0.648	0.670	0.784	0.940		
Trust	0.757	0.881	0.774	0.677	0.731	−0.776	0.611	0.634	0.637	0.645	0.673	0.871	0.950	
Use of emotion	0.817	0.884	0.764	0.856	0.744	−0.795	0.721	0.612	0.618	0.639	0.645	0.786	0.756	0.947

### Assessment of structural model

There are three main evaluation metrics for assessing the robustness of structural models: the collinearity concerns test for the structural model, the coefficients of determination (R2) and their statistical significance explain the variance.

### Multicollinearity assessment

For the first step, we measured the VIF and tolerances of the structural model in order to find out if it was multicollinear. Based on the study material and rules of thumb, we were able to conclude that there was no collinearity issue in Table 4 since both values fell inside the range that is indicated by a tolerance value more than 0.2 and a VIF value less than 5, as stated by Hair et al. (2014).

**TABLE 4:** Second-order measurement model.

Constructs	SD	Weights	*T*-values	Loadings	*T*-values
Integ–> Org Virt	0.038	0.220	5.776	0.989	781.8
Comp–> Org Virt	0.031	0.335	10.703	0.989	699.8
Trust–> Org Virt	0.029	0.180	6.182	0.893	562.3
Optim–> Org Virt	0.040	0.154	3.870	0.918	33.6
Forg–> Org Virt	0.030	0.124	4.177	0.985	659.0
SEA–> EI	0.043	0.419	9.664	0.955	1346.8
OEA–> EI	0.044	0.384	8.795	0.994	1272.9
UOE–> EI	0.047	0.045	0.338 (NS)	0.989	655.7
ROE–> EI	0.043	0.158	3.667	0.992	959.8
PWB–> WB	0.033	0.320	9.639	0.997	2888.8
NWB–> WB	0.031	0.383	12.187	0.996	2135.4
SWL–> WB	0.030	0.300	10.168	0.995	1682.1

### Coefficients of determination R^2^ and hypothesis testing

An endogenous construct’s predictive ability was measured by its R2 value (coefficient of determination) in the research model by Henseler et al. (2009). It was then determined that there was a level and significance associated with the path coefficient so that the PLS algorithm could be run to test the hypothesis and bootstrapping could be done. As can be seen in Table 5, the study’s R2 findings can be summarized as follows.

**TABLE 5:** Multicollinearity.

Variable	Level of tolerance	VIF
ORG VRT	0.433	2.058
EI	0.537	1.862
WB	0.263	3.798

As a rule of thumb, Henseler et al. (2009) recommend R2 values of 0.75, 0.50, and 0.25 for endogenous latent constructs relate to substantial, moderate, and weak constructs, respectively. 94.4% of the variance in the WB was accounted for by independent variables, including OV and EI, as evidenced in Table 6. There was also a substantial R2, with a value of 86.5 and 88.4% for the R2 of ERP and IRP, respectively.

**TABLE 6:** Variance explained by exogenous constructs.

Constructs	Variance explained (R2)	Level
WB	94.40%	Substantial
IRP	86.50%	Substantial
ERP	88.40%	Substantial

**FIGURE 5:**
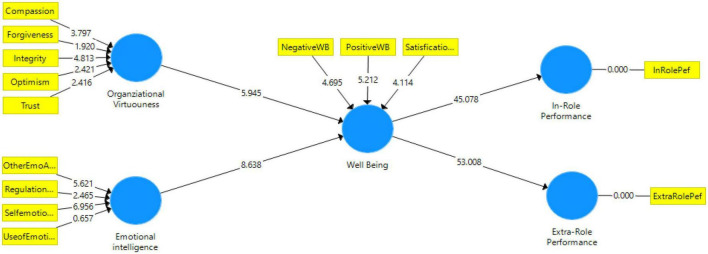
Structural model assessment.

We took certain steps according to the guidelines that were described in Preacher and Hayes (2004) in order to test the mediation hypothesis, which is a path coefficient, in this study. As a result of this, bootstrapping was performed in the PLS-SEM model based on these assumptions. To determine the “*P*-value” and “*T*-Value” of the bootstrapping process, two-tailed tests of significant level 5% were run in Smart-PLS, followed by 2-tailed test significance. Hence, if a *t*-value of 1.96 is considered to be a critical value, as a result, this should serve as a threshold value in our current study when evaluating the hypotheses. The correlation coefficient for structural models along the measurement path can be seen in Figure 5.

The following table gives the results of the structural model, which are shown in Table 7. As it turns out, the first hypothesis H1 suggests that “wellbeing mediates the relationship between OV and IRP” at a level of significance of 0.05 was significant (β = 0.367, *t* = 5.925, *p* < 0.01). Based on the results of the analysis, the second hypothesis H2, which states that wellbeing mediates the correlation between OV and ERP, has also been confirmed (β = 0.370, *t* = 5.906, *p* < 0.01). There was also a significant correlation between EI and IRP when H3 was assumed to be the mediator (β = 0.539, *t* = 8.293, *p* < 0.01). In the final hypothesis H4 we also found that an association between EI and ERP is mediated by wellbeing (β = 0.545, *t* = 8.387, *p* < 0.01). It can be concluded that the proposed hypotheses of the study were all supported by the findings of the study.

**TABLE 7:** Hypothesis result.

	Hypo	Beta	SD	*T*-value	*P*-values	Results
H1	Org Virt_–> WB–> IRP	0.367	0.062	5.925	0.000	Supported
H2	Org Virt_–> WB–> ERP	0.370	0.063	5.906	0.000	Supported
H3	EI–> WB–> IRP	0.539	0.065	8.293	0.000	Supported
H4	EI–> WB–> ERP	0.545	0.065	8.387	0.000	Supported

## Discussion

With the introduction of COVID-19, the fragile state of young people’s mental health and wellbeing around the world has only become more precarious, which is particularly alarming when we consider the already precarious state of young people’s mental health and wellbeing. The urgency of conducting high-quality research to address mental health problems has multiplied by several orders of magnitude following a pandemic of COVID-19’s magnitude. Based on these consideration, the study developed a research framework and examined the work related subjective wellbeing as a process underlying the associations between positive feature of work environment (OV) with IRP, between positive feature of work environment (OV) with ERP, between individual factor (EI) with IRP and between individual factor and ERP. The study found that Perception of OV impact both IRP and ERP through wellbeing. The significant mediating role of WEB between OV and job performance is consistent with tenants of OST (Eisenberger et al., 1986) and AET (Weiss and Cropanzano, 1996).

From the OST perspective, within a working environment characterized by organizational virtuousness, employees perceive that their organization cares about them by forgiving their mistakes, treats them well with compassion, values them by trusting them, and gives them respect. Thus, employees who perceive organizational virtuousness experience an increased level of wellbeing. With this feeling of gratitude for working in a virtuous organization, the individuals feel compelled to reciprocate with acts that benefit the organization and other people. It means that individuals who perceive their organizations as virtuous develop positive feelings of attachment with the virtuous agents; such feelings may increase their wellbeing, thus reacting by exhibiting higher levels of productivity, greater job performance and behaviors that may also go beyond their in-role duties.

Furthermore, Affective events theory (Weiss and Cropanzano, 1996) also helps to understand and support the mediating relationship. This theory suggests that stable work environments influence the occurrence of positive and negative affective events. Experiencing these events leads employees to experience affective states. Affective states, in turn, derive behavior. In incoherence with such theory, working in a virtuous context is an important affective event, thus triggering higher wellbeing and leading to derive IRP and ERP.

According to a few previous studies, perceptions of OV also contribute to WEB and subsequent job performance (e.g., Ahmed et al., 2018; Magnier-Watanabe et al., 2018; Setyoko and Kurniasih, 2022; Sun and Yoon, 2022) in different context. For example, Sun and Yoon (2022) argued that an organization with virtuous actions produces social and individual happiness. When employees are happy, it eventually leads to such complacency, leading to extra-role behaviors. Similarly, Magnier-Watanabe et al. (2018) also confirmed the importance of wellbeing on employees’ job performance. They suggested that the firms pay attention to their organizational virtuousness because it promotes higher wellbeing, which in turn enhances job performance in this perspective. The findings are also consistent with Pimentel et al. (2022) suggesting that positive organizational features may influence extra-role performance because organizational identification increases, leading to feelings of happiness which, in turn, induce employees to perform extra-role behaviors that benefit themselves and the organization.

In addition, the results of this study demonstrated that a person’s level of WEB plays an important mediating role in the relationships between individual factors (EI) and both IRP and ERP. The significant mediating role of wellbeing between EI and job performance is consistent with the Affective Events Theory. The theory posits that an individual dispositional variable (EI) can have both a direct and indirect effect on a wide range of employee work behaviors, including IRP and citizenship behaviors (Weiss and Cropanzano, 1996; Greenidge et al., 2014; Vratskikh et al., 2016). Theoretical tenets underlying the notion of AET suggested that various affective (emotional) and attitudinal factors serve as key mediating mechanisms that emerge to facilitate or hinder employees’ work behaviors (e.g., job performance) (Weiss and Cropanzano, 1996). Under the framework of AET, Wellbeing (which comprises both affective and attitudinal dimensions) is considered a critical intervening mechanism in the relationship between dispositional factors (e.g., EI or personality traits) and behavioral outcomes at work.

The finding is also in accordance with limited past studies that had examined the mediating role of wellbeing in the different contexts of usage (e.g., Spector and Fox, 2002; Joseph and Newman, 2010; Brunetto et al., 2012; Devonish, 2016; Li et al., 2018). For example, Joseph and Newman (2010) postulated that in the organizational setting, EI is theoretically related to job performance through the induction of affective states (wellbeing) that are beneficial to job performance. Devonish (2016) argued that individual difference variables (EI) indirectly affect the IRP and ERP of employees through different affective, attitudinal, and emotional mechanisms.

Also, Miao et al. (2017) found that EI helped employees to cope with negative emotions that may lead to improper behaviors in the workplace. Since managing emotions is a key part of EI, individuals facilitate their behavior by adjusting their emotions. Employees with higher EI coped better with various matters at the workplace and tend to experience more wellbeing, leading them to engage in positive work behaviors. Similarly, Brunetto et al. (2012) demonstrated that EI positively predicted wellbeing, which, in turn, promoted employee engagement. Li et al. (2018) also considered wellbeing as the mediator between EI and job performance. This study revealed that EI affects teachers’ job performance through employee wellbeing. In sum, all these studies have established EI’s role in enhancing IRP and ERP through promoting wellbeing.

In conclusion, these findings suggest that employees who scored higher on the individual dispositional scale were better able to deal with issues that arose in the workplace and tended to be happier and more fulfilled, both of which drove their IRP and ERP behavior while they were on the job. Furthermore, the findings of this study are in stark contrast to the assumptions of the AET. An employee’s EI is considered a dispositional variable, meaning it can affect him or her directly or indirectly, depending on the situation. This is one of the many ways in which the results are supported theoretically (Greenidge et al., 2014). By relying on the assumption that wellbeing mediates the relationship between EI and job performance, the study contributed to the expansion of AET, which had not been examined under a tenant of the theory in the past.

## Conclusion

Evidence is growing that the employee wellbeing and their general welfare is the key factor at the workplace for causing difference in the individual and organizational level performance. As a consequence of this, the concept of wellbeing as measured by levels of pleasure, satisfaction, and happiness has emerged as an essential topic of investigation in the field of organizational research. In addition, the findings of the study demonstrated that the academic idea of wellbeing takes on a significant role in the context of its application in the context of the workplace when it is conceived of as a notion that is unique to that area. This study empirically proved the components of wellbeing. In relation to working, SWBs have been largely ignored to date. Furthermore, academicians from diverse disciplines are unsure how to operationalize the concept of wellbeing, whether it should be understood as merely attitudinal, affective, cognitive, or both (affective and cognitive). According to the findings of an in-depth analysis of the pertinent literature, several different terms are regularly interchanged with one another in the context of work, including “wellbeing,” “job satisfaction,” and “happiness,” contributing to the confusion as to the exact meanings of these terms. Toward understanding this conceptualization of individual wellbeing, the model of subjective wellbeing that Diener (1994) put up, which includes both cognitive and affective dimensions, has gained widespread acceptance. In light of this, and in accordance with the suggestions made by earlier researchers (e.g., Bakker and Oerlemans, 2011; Magnier-Watanabe et al., 2017, 2020), and to further avoid any confusion and misinterpretation, which previous scholars have made in examining work-related wellbeing in relation to the job performance, this study proposed wellbeing as a multidimensional construct and measured it as high-order composite wellbeing, with an affective and cognitive component on the lower order. This model of workplace SWB was shown to be validated by empirical evidence and sound theoretical approach, while it still requires additional research to provide additional empirical support. Therefore, work related subjective wellbeing is proved to be certain type of attitude. This is an important finding. In the light of study findings it is concluded that an employee at the work place is said to be happy and high in wellbeing when he is feeling more positive emotions, less negative emotions and satisfied with his work life domain. Therefore, employee this attitude will result in high job performance. Thus, in order to make them happy, organizations needs to focus on internal and external factors that will create happiness at the workplace.

In this study, decades of confusion were cleared up, revealing that subjective wellbeing will be key to organizational performance and growth in the future. The psychological mechanism in explaining the several links among workplace variables can be better understood with the findings of the study by considering work related subjective wellbeing as a mediating variable. Likewise, by distinguishing IRP and ERP as two different types of performances, the study succeeded in highlighting the merit in understanding the importance of both the performances especially in the banking sector. It is important to progress in developing the theory of workplace happiness and extension of happy worker thesis. In this notion, this study is an important attempt to resolve the holy grail of organizational psychology in management by extending the concept of subjective wellbeing to the workplace. Further, overall result also provided useful insight for the management of the banks to focus on the factors that can make employees happy and productive at the workplace. More importantly, to deal with the COVID-19 pandemic threats that adversely affect the performance of employees at banks, the research findings concluded that it can also be addressed by focusing on the same factors that triggers the wellbeing and happiness of employee at the workplace which will ultimately resulted in enhancing their performance even in the tough times.

## Limitations and future research

Despite the fact that the research spans a wide scope of territory to be investigated, it is nonetheless subject to a few limitations. The fact that the research was conducted using a cross-sectional design and five different banks also raises the possibility of common method variance, and generalizing the result to whole banking sector. In addition, the IRP and ERP used in this study are self-reported, thus they may over- or under-estimate an individual’s achievement relative to their true potential. Furthermore, future researchers could get better outcomes by concentrating on and creating a unified scale to evaluate subjective wellbeing in the workplace. Considering the correlation between independent variables and dependent variables can provide us with a better understanding of the phenomenon, the results could be replicated in other context and industries. In addition, studies in the future could investigate the moderating variable in the research model. Furthermore, the study findings can be used as guiding principles for other researchers from the field of organizational behavior, business management, psychology, and the positive psychology movement interested in developing the theory of workplace happiness. While most researchers focusing only on the social exchange theories and Job demand resources in understanding workplace variables relationship, the theory of Happiness at the workplace needs to be build up that have been ignored for decades despite its importance and generally acceptability in the organizational setting.

## Data availability statement

The raw data supporting the conclusions of this article will be made available by the authors, without undue reservation.

## Author contributions

MA, DA, and NZ conceptualized the model. MA and DA wrote the manuscript. MA collected the data. NZ analyzed the manuscript and proof read the draft. All authors contributed to the article and approved the submitted version.
